# Antidiabetic Properties of *Azardiracta indica* and *Bougainvillea spectabilis*: *In Vivo* Studies in Murine Diabetes Model

**DOI:** 10.1093/ecam/nep033

**Published:** 2011-06-18

**Authors:** Menakshi Bhat, Sandeepkumar K. Kothiwale, Amruta R. Tirmale, Shobha Y. Bhargava, Bimba N. Joshi

**Affiliations:** ^1^Institute of Bioinformatics and Biotechnology, India; ^2^Department of Zoology, University of Pune, Pune, India

## Abstract

Diabetes mellitus is a metabolic syndrome characterized by an increase in the blood glucose level. Treatment of diabetes is complicated due to multifactorial nature of the disease. *Azadirachta indica Adr. Juss* and *Bougainvillea spectabilis* are reported to have medicinal values including antidiabetic properties. In the present study using *invivo* diabetic murine model, *A. indica* and *B. spectabilis* chloroform, methanolic and aqueous extracts were investigated for the biochemical parameters important for controlling diabetes. It was found that *A. indica* chloroform extract and *B. spectabilis* aqueous, methanolic extracts showed a good oral glucose tolerance and significantly reduced the intestinal glucosidase activity. Interestingly, *A. indica* chloroform and *B. spectabilis* aqueous extracts showed significant increase in glucose-6-phosphate dehydrogenase activity and hepatic, skeletal muscle glycogen content after 21 days of treatment. In immunohistochemical analysis, we observed a regeneration of insulin-producing cells and corresponding increase in the plasma insulin and c-peptide levels with the treatment of *A. indica* chloroform and *B. spectabilis* aqueous, methanolic extracts. Analyzing the results, it is clear that *A. indica* chloroform and *B. spectabilis* aqueous extracts are good candidates for developing new neutraceuticals treatment for diabetes.

## 1. Introduction

Diabetes is a chronic disorder affecting the population on epidemic level. Diabetes results from abnormal metabolism of insulin wherein insulin action is impaired, or absolute insulin deficiency results in imbalance of glucose metabolism and leads to a syndrome called diabetes mellitus [1, 2].

In the present study using *invivo* streptozotocin (STZ)-induced diabetic murine model, we investigated the antidiabetic properties of different extracts of *A. indica* and *B. spectabilis* and tested biochemical parameters such as fasting glucose level, glucosidase activity, G6PD activity, hepatic and skeletal glycogen stores and restoration of functional *β*-cell mass for possible biological action.

Many oral antidiabetic drugs used today fail to give a long-term glycemic control [3, 4]. The herbal extracts which are effective in lowering blood glucose level with minimal or no side effects are known to be used as antidiabetic remedies [5, 6]. Many compounds isolated from these plants are used in combinational therapy for diabetes [7–12]. Herbal extracts from *Tamarindus indica*, *Ceilba pentandra, Syzygium cuminii* and *Trigonella foenum* are known for their hypoglycemic effects [13–15]. *Azadirachta indica Adr. Juss*. is known to possess hypolipidemic, hypoglycemic, immunostimulant and hepatoprotective properties [16, 17]. While nimbocinone, nimolinone, kulactone, nimocinolides, isonimocinolide, nimbin, salanin, azadirachtin, flavonoids, myricetin, meldenindiol, vilasinin, margosinolide, isomargosinolide, desacetyldihydronimbic acid have been isolated from *A. indica* leaves having medicinal properties [18–21], *Bougainvillea spectabilis* leaves contain d-pinitol (3-*O*-methylchiroinositol) and is claimed to exert insulin-like effect [22–24]. We have earlier confirmed the glucosidase inhibitory activity of *A. indica* and *B. spectabilis* against murine pancreatic and intestinal glucosidase [25], suggesting one of the important underlying mechanisms of antidiabetogenic activity.

There are reports showing plant extracts exerting beneficial effects in the diabetic environment by improving and/or mimicking insulin action [26, 27]. They are also thought to enhance insulin secretion in streptozotocin-induced diabetic mice [7, 28]. The insulin deficiency causes changes in glucose metabolism and biochemical processes, thereby increasing fasting glucose level, decreasing hepatic and skeletal glycogen content and decreasing glucose-6-phosphate dehydrogenase (G6PD) activity. Reduction in G6PD activity lowers the intracellular NADPH level that causes oxidative stress, a critical underlying mechanism in diabetes [29]. During prolonged diabetic condition, insulin resistance results in the depletion of glycogen stores in liver and tissue muscles which leads to hyperglycemia and weight loss [30]. One of the common complications seen in diabetic patients is postprandial hyperglycemia (PPHG), and therapeutic approaches for decreasing PPHG is to retard absorption of glucose by the inhibition of carbohydrate-hydrolyzing enzymes, namely, glucosidases [31, 32] Figure 7.

## 2. Materials and Methods

### 2.1. Plant Material

The leaves of *A. indica* (herbarium no MBBP 5) and *B. spectabilis* (herbarium no MBBP 1) were collected from the Western Ghats in and around Pune city in the months of May and June, and were authenticated by the Botanical Survey of India (BSI), Pune. The plant material was sequentially extracted through a soxhlet extractor using methanol, chloroform and distilled water [25].

### 2.2. Toxicity Bioassay

The toxicity test of extracts was carried out on brine shrimp, *Artemia salina* [33]. Brine shrimp eggs were hatched in artificial sea water prepared from 20% sea salt (Sigma Aldrich, USA). After 48 h of incubation (at 25–29°C), nauplii (larvae) were collected in 96-well microtiter plate. Ten larvae were added in each well containing serially diluted crude plant extracts. After 24 h of incubation, live and dead larvae were counted and LC_50_ was determined for each plant extract [34].

### 2.3. Induction of Diabetes Mellitus

The *invivo* study was conducted on Swiss mice (6–8 weeks), weighing 25 ± 2 g, at an ambient temperature of 25 ± 2°C and standard food and water *ad libitum*. The mice were made diabetic with a single intraperitonial injection of STZ (3 mg/25 g of body weight) in citrate buffer, pH 4.5 [35]. This dose produced diabetes after 7 days with blood glucose level of approximately (200–250) mg/dL. The entire procedure was carried out as per stated guidelines of Institutional Animal Ethical Committee.

### 2.4. Treatment with Plant Extracts

Plant extracts (100 *μ*g/200 *μ*L) reconstituted in distilled water with 0.5% DMSO were injected intraperitonially into the experimental mice for 21 days. Six animals were kept in each group and the non-diabetic control and diabetic control received vehicle (distilled water + 0.5% DMSO) only. After 21 days of the treatment, the animals were sacrificed, organs were removed, washed with PBS and stored at –20°C for further use.

### 2.5. Fasting Blood Glucose Level and Weight of Animals

The fasting glucose level was monitored periodically during the treatment with the tail prick method using Accucheck Active Glucometer (Roche Diagnostics GmbH, Germany). The blood glucose level was measured in milligram per deciliter. The weights of the animals were checked before and after treatment.

### 2.6. Oral Glucose Tolerance Test (OGTT)

The oral glucose tolerance test was performed on overnight fasted normal, diabetic and treated mice on 21st day of the treatment. Glucose (2 g/kg body weight) was given orally and blood glucose level was measured at 0, 30, 60, 90, and 120 min after administration of glucose [36].

### 2.7. Pancreatic and Intestinal Glucosidase Activity

The mice were sacrificed after 21 days of treatment. Pancreatic and small intestinal tissues were homogenized in 10 mM PBS containing 100 mM NaCl and protease inhibitor cocktail. The homogenates were centrifuged and the supernatant was used as a source of enzyme. The glucosidase activity was measured by DNSA method [25].

### 2.8. G6PD Activity

G6PD activity was checked by Langdon method [37]. The liver tissue posttreatment was homogenized in 10 mM PBS. The change in absorbance of reaction mixture containing 1 M Tris-chloride buffer, 25 mM glucose-6-phosphate, 0.2 M magnesium chloride, 2 mM NADP and homogenized tissue was measured at 340 nm for 10 min. One unit of enzyme activity was defined as the quantity which catalyzed the reduction of 1 *μ*M of NADP per minute [13].

### 2.9. Glycogen Estimation

Glycogen was estimated from liver and muscle tissues post treatment [38]. The tissues were homogenized in warm ethanol and centrifuged. The residue was dried and dissolved in distilled water with perchloric acid for extraction. This mixture was centrifuged and the supernatant was estimated for glucose concentration using phenol sulphuric acid method at 490 nm. The amount of glycogen in the tissue sample was expressed in micrograms of glucose per milliliter of tissue extract [13].

### 2.10. Immunohistochemical Analysis

Pancreas from control diabetic and treated mice were fixed in 4% paraformaldehyde. Seven-micrometer thick sections were taken on a rotary microtome (Thermo Scientific, Shandonfinsse) and used for immunohistochemical analysis.

### 2.11. Western Blot Analysis

Equal amount of pancreatic proteins from control diabetic and treated mice were separated on 10% SDS polyacrylamide gel and transferred to nitrocellulose membrane. The human recombinant insulin was used as positive control. These blots were probed using guinea pig anti-insulin antibody and the reaction was visualized using 3,3-diaminobenzidine tetrahydrochloride. The densitometric analysis was performed on the developed immunoblot to quantify insulin secretion.

### 2.12. Plasma Insulin and C-Peptide Analysis

The plasma insulin and c-peptide were quantitated using mouse insulin (Mercodia, Sweden) and c-peptide (Yanaihara Institute, Japan) Elisa kit.

### 2.13. Statistical Analysis

Data was evaluated using Student's paired *t*-test, one way ANOVA or two-way ANOVA analysis where appropriate. Groups were considered to be significantly different if *P* ≤  .05.

## 3. Results

### 3.1. Toxicity Analysis

The toxicity of extracts of *A. indica* and *B. spectabilis* was tested by brine shrimp bioassay. The average LC_50_ for *A. indica* was 7.5 mg/mL and *B. spectabilis* was 10 mg/mL. The total dose of plant extracts given to the treatment group over a period of 21 days was 2 mg/4 ml which is much lower than the LC_50_ value.

### 3.2. Fasting Glucose Level and Weight of Animals

The fasting glucose level was periodically monitored during the treatment. It was found that chloroform extract of *A. indica* and aqueous, methanolic extracts of *B. spectabilis* showed a gradual decrease in fasting glucose level over the period of 21 days. There was a significant weight gain comparable to the control, indicating an increase in muscle mass (Table 1).

### 3.3. OGTT

**Figure 1:**
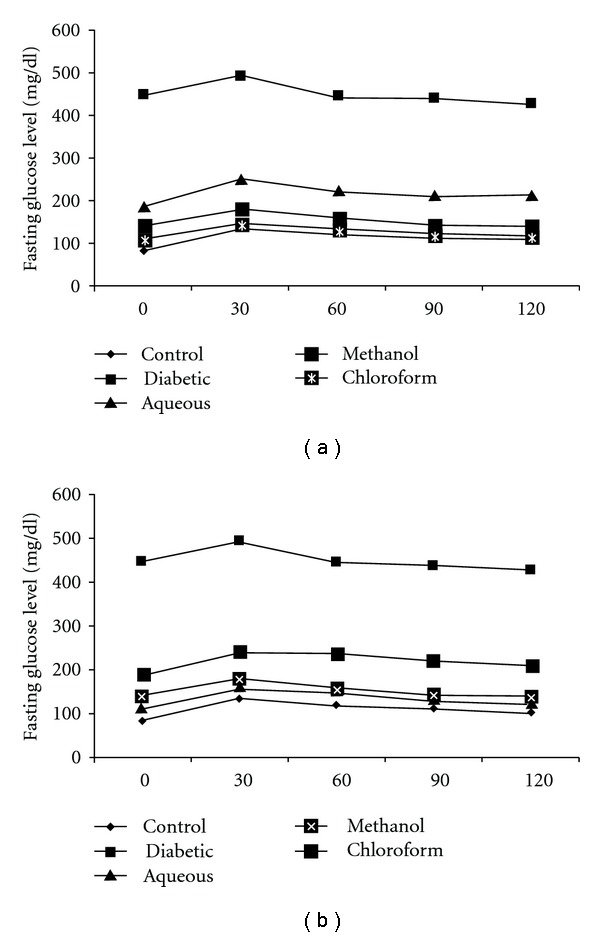
The data indicates the oral glucose tolerance after the treatment with (a) *A. indica* and (b) *B. spectabilis.* The data are indicated as mean ± SEM (*n* = 6).

The glucose tolerance test was performed to check the glycemic control of plant extracts after the treatment. *A. indica* chloroform extract and *B. spectabilis* aqueous, methanolic extracts show good oral glucose tolerance (Figure 1).

### 3.4. Pancreatic and Intestinal Glucosidase Activity

**Figure 2:**
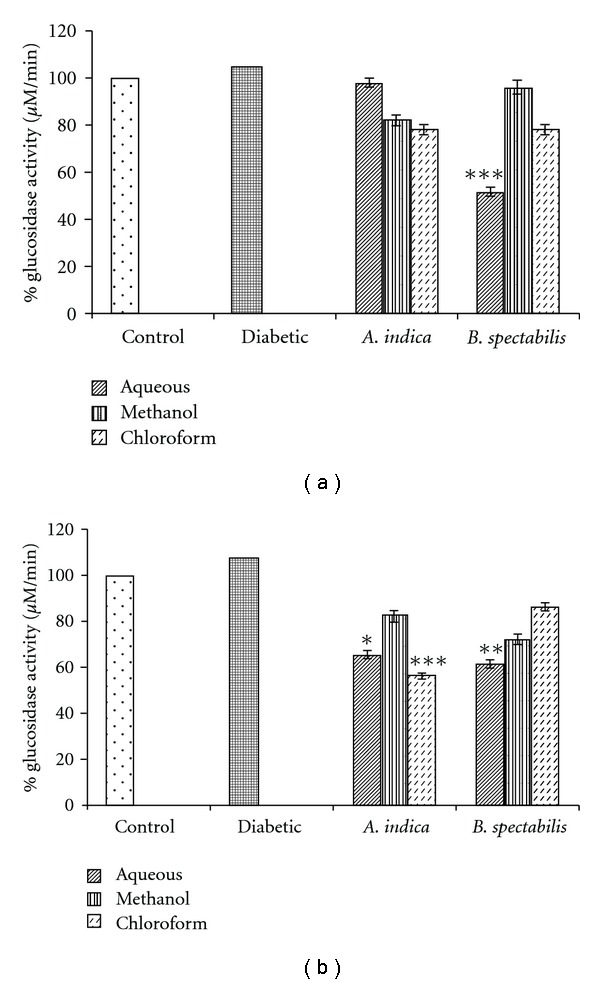
Indicates post-treatment (a) pancreatic, and (b) intestinal glucosidase activity. Data was calculated as mean ± SEM (*n* = 6). The student *t*-test was used where bars with different asterisks (***, **, *) shows a significant difference with respect to control where *P* <  .05.

The chloroform and aqueous extract of *A. indica* showed a significant decrease in the enzyme activity of intestinal glucosidase 51% and 35%, whereas there was no significant reduction in pancreatic glucosidase. *B. spectabilis* aqueous extract showed inhibition with intestinal as well as pancreatic glucosidase of 52% and 48%, respectively (Figure 2).

### 3.5. G6PD Activity

**Figure 3:**
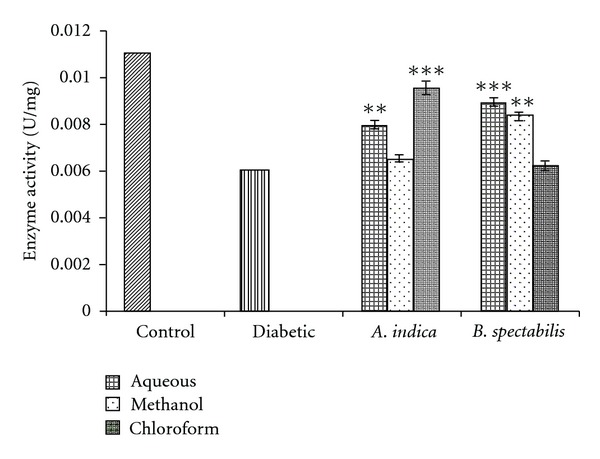
Indicates G6PD restoration activity of plant extract post-treatment. Data was calculated as mean ± SEM (*n* = 6). The student *t*-test was used where bars with different asterisks (***, **) shows a significant difference with respect to control where *P* *<*  .05.

G6PD indicates the proper glucose utilization through pentose phosphate pathway in the liver. The diabetic mice showed a 50% reduction in G6PD activity in the liver as compared to control after 21 days. There was a significant increase in G6PD activity with *A. indica* chloroform, aqueous and *B. spectabilis* aqueous, methanolic extracts after 21 days of treatment (Figure 3).

### 3.6. Liver and Muscle Glycogen Content

**Figure 4:**
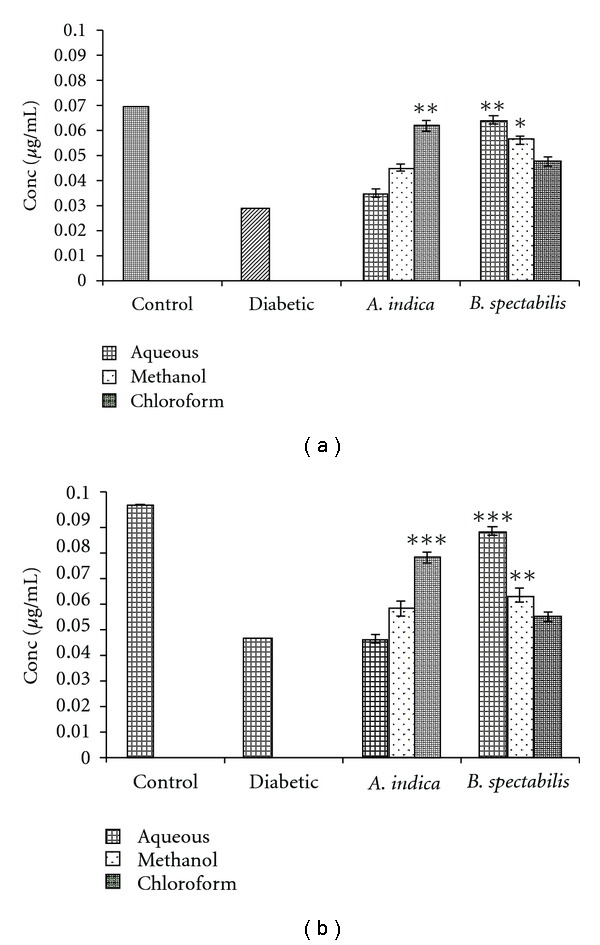
Indicates glycogen storage of (a) liver and (b) skeletal muscle post-treatment. Data was calculated as mean ± SEM (*n* = 6). The student *t*-test was used where bars with different asterisks (***, **, *) shows a significant difference with respect to control where *P* *<* .05.

Hepatic and skeletal muscle glycogen content was estimated post treatment. In the case of diabetic mice, hepatic and muscle glycogen content was reduced up to 45%. The chloroform extract of *A. indica* and aqueous, methanolic extracts of *B. spectabilis* showed significant increase in liver and muscle glycogen content (Figure 4).

### 3.7. Immunohistochemical and Western Blot Analysis

**Figure 5:**
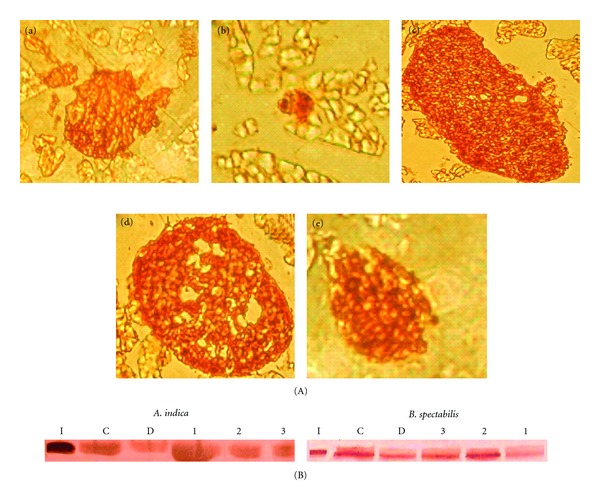
(A) Immuno-histochemical analysis of the mouse pancreas developed with anti-insulin antibody. (a) Normal, (b) Diabetic, (c) *A. indica* chloroform extract treated, (d) *B. spectabilis* aqueous extract treated, (e) *B. spectabilis* methanolic extract treated with magnification of 480x, and (B) immunoblot analysis of pancreatic protein probed with anti-insulin antibody after treatment with (I) Human recombinant insulin, (C) control, and (D) diabetic (1) chloroform (2) methanolic (3) aqueous. The data are measured by densitometry in ODU/mm^2^.

Immunohistochemical and western blot analyses were performed to quantify insulin production and *β*-cell function post treatment. The chloroform extract of *A. indica* and methanolic, aqueous extracts of *B. spectabilis* showed increase in functional islets actively producing insulin. Densitometric analysis of western blot resulted in comparable insulin production as seen in immunohistology.  Figure 5(A) shows representative picture of pancreatic sections and Figure 5(B) represents immunoblot developed on same pancreatic tissue protein.

### 3.8. Plasma Insulin and C-Peptide Analysis

**Figure 6:**
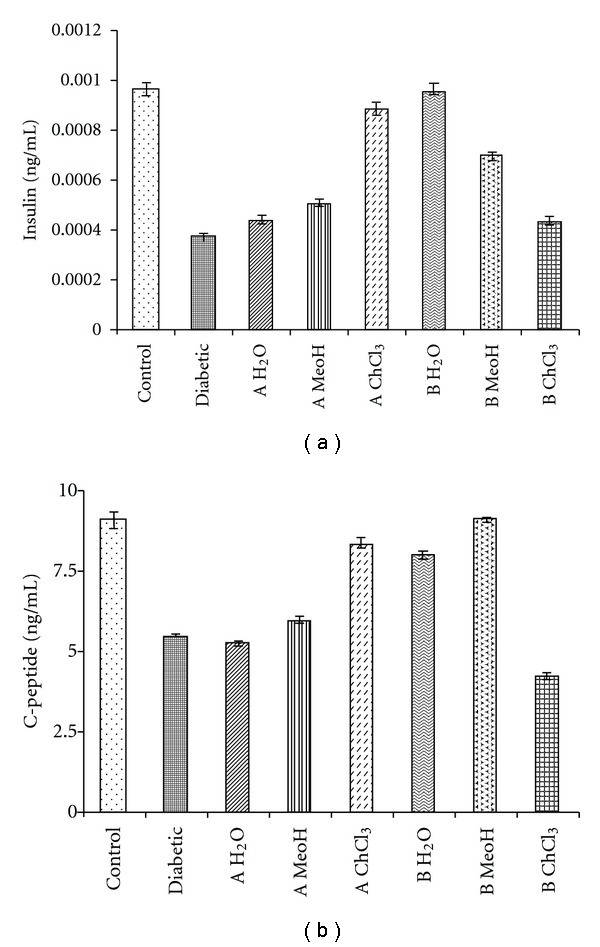
Indicates the insulin and c-peptide analysis using Elisa kits.

Plasma insulin and c-peptide were analyzed to check the proper endogenous insulin secretion from *β*-cells after treatment with plant extracts. Insulin and c-peptide were measured in nanograms per milliliter. Again the chloroform extract of *A. indica* and aqueous, methanolic extract of *B. spectabilis* was able to normalize the plasma insulin and c-peptide levels (Figure 6).

## 4. Discussion

*A. indica* and *B. spectabilis* are known for their antidiabetogenic activities. The earlier studies of these plant extracts on diabetic rats have shown prevention of diabetes and oxidative stress [39, 40]. In the present study, we report the effect of *A. indica* and *B. spectabilis* extracts on biochemical parameters important with respect to diabetes in *invivo* murine model. The effect of plant extracts on metabolic pathways in STZ-induced diabetic mice was studied in a duration-dependent manner. STZ is known to produce hyperglycemia by selective cytotoxic effect on pancreatic *β*-cells [41]. A single intraperitonial injection of STZ given to normoglycemic mice produced hyperglycemia in 7 days with blood glucose level of 200–250 mg/dL.

It was observed that treatment of chloroform extract of *A. indica* and aqueous, methanolic extracts of *B. spectabilis* for 21 days reduced the fasting glucose to significantly normal level, acquiring good glycemic control suggesting its antihyperglycemic properties (Figure 1). The onset of antihyperglycemic effect with these extracts was seen within 10 days of treatment, even though maximum and significant reduction was observed only after 15 days. Since we did not observe any more changes in fasting blood glucose, the experiment was terminated on 21st day. Body weight, which is another important parameter in diabetes, also increased with the treatment of *A. indica* chloroform extract and *B. spectabilis* aqueous extract (Table 1). There was a gradual increase in the body weight of controls and treated mice while the diabetic mice continued to lose weight.

**Figure 7:**
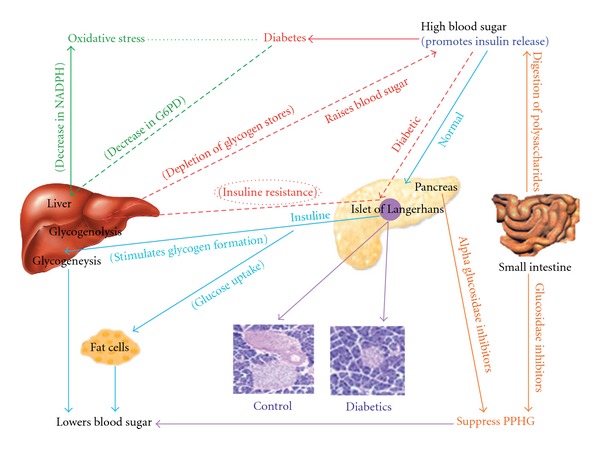
Diagrammatic representation of glucose metabolic pathways in normal and diabetic case.

In our earlier study, *A. indica* is reported as a pancreatic, and *B. spectabilis* as an intestinal glucosidase inhibitor [25]. This was also evident in STZ-induced diabetic mice wherein reduction of pancreatic and intestinal glucosidase was observed after supplementing these extracts. Glucosidase are normally present in the pancreas and gut of small intestine and play an important role in the digestion of dietary carbohydrates. The inhibitors of *α*-glucosidase, in consequence retard the use of dietary carbohydrates to suppress PPHG in diabetes [42, 43] Figure 7. The chloroform extract of *A. indica* and methanolic, aqueous extracts of *B. spectabilis* retarded the glucosidase activity which helps in controlling PPHG, resulting in reduction of blood glucose level (Figure 2). Interestingly, *A. indica* chloroform extract has shown 50% reduction in intestinal glucosidase, which was not seen during an *invitro* enzyme inhibition assay.

In an attempt to gain an insight into the underlying biochemical mechanisms involved in antidiabetogenic activities of plant extracts, G6PD activity was estimated. G6PD is the key enzyme in pentose phosphate pathway which helps in maintaining the normal blood glucose levels [44] (Figure 7). In STZ-induced diabetic murine model, G6PD activity in liver reduces significantly which obstructs glucose utilization and leads to hyperglycemia [45]. The treatment with chloroform extract of *A. indica* and aqueous, methanolic extracts of *B. spectabilis* resulted in a significant restoration of the enzyme activity in treated mice which could be due to increase in insulin production as this enzyme activity depends on insulin (Figure 3).

Insulin is the main regulator of glycogen synthesis in hepatic and skeletal muscles (Figure 7). It promotes glycogen synthesis by stimulating glucose uptake and activating the key enzyme, glycogen synthase [30, 46]. There are reports of impairment of hepatic and skeletal glycogen synthesis in diabetic rats [47–49]. We observed 50% reduction in both muscle and hepatic glycogen content in diabetic mice which may be due to lack of insulin in the diabetic state. The supplementation of *A. indica* chloroform extract and *B. spectabilis* aqueous and methanolic extracts has shown a significant increase in glycogen level in diabetic mice which may be due to the reactivation of glycogen synthase systems (Figure 4). This indicates that plant extracts have a direct effect on muscle and liver glucose metabolism which helps in prevention of loss of muscle mass in diabetes.

In the present study, we further performed immunohistochemical analysis of pancreatic sections to check insulin secretion and islet regeneration properties of these plant extracts. It was found that *A. indica* chloroform and *B. spectabilis* aqueous, methanolic extracts showed significant regeneration property of functional *β*-cells (Figure 5(A)). The immunoblot and densitometric analysis on pancreatic protein further supported the histological finding showing increase in insulin levels with these extracts (Figure 5(B)). It is known that there is decrease in c-peptide levels during diabetes because of autoimmune destruction of *β*-cells. In our study, we measured insulin and c-peptide levels from treated animals to determine how much of their own natural insulin is being produced. We observed normalization in plasma insulin and c-peptide levels which further corroborated the formation of functional islets and insulin release with *A. indica* chloroform extract and *B. spectabilis* aqueous extracts treatment. Earlier, *Gymnema sylvestry* was reported to have pancreatic islet regeneration property within diabetic mice [50]. This further supports the data that *A. indica* chloroform extract and *B. spectabilis* aqueous extracts have islet regenerative and insulin-producing property. These extracts stimulate the *β*-cells to secrete insulin in STZ-induced diabetic mice, resulting in the improvement of carbohydrate metabolism towards the re-establishment of normal blood glucose level.

This study is significant as it covers various important biochemical and metabolic aspects responsible for the progression of diabetes. These plant extracts have definitely shown multiple biological targets; however, at this stage it is difficult to predict whether all the components act independently or in synergetic manner because active principles or biomolecules are responsible for their antidiabetogenic effect, which is required to be identified. One common reason emerging from this study is the effect of the extracts on islets regeneration and insulin production which can indirectly modulate all other biochemical parameters. Due to the interesting finding of possible islet regenerative/protective property and low toxicity, these plant extracts can be evaluated further for their introduction as neutraceutical for the treatment of diabetes.

## 5. Conclusion

The data suggest that *A. indica* chloroform extract and *B. spectabilis* aqueous, methanolic extracts exhibit islet regeneration/protection properties and therefore have beneficial effects in diabetes mellitus that holds the hope of new generation of antidiabetic drugs.

## Funding

Senior Research Fellowship from Council of Scientific and Industrial Research (CSIR, Government of India) (to M.B.); financial support from CSIR, India.

## Figures and Tables

**Table 1:** Effect of plant extracts on fasting glucose levels and weight of the animals after 21 days of treatment. The data is indicated as mean ± SEM; (*n* = 6).

Group (*n* = 7)	Fasting glucose level (mg/dL)	Body weight (g)	Body weight (g)
Diabetic	462 ± 6.22	19 ± 8.66
Control	85 ± 6.54	31 ± 7.66
*Azadirachta indica* (H_2_O)	187 ± 2.03	21 ± 7.36
*Azadirachta indica* (MeOH)	140 ± 2.43	27 ± 11.36
*Azadirachta indica* (ChCl_3_)	109 ± 5.65	30 ± 4.25
*Bougainvillea spectabilis* (H_2_O)	112 ± 5.22	30 ± 5.59
*Bougainvillea spectabilis* (MeOH)	120 ± 10.26	28 ± 6.98
*Bougainvillea spectabilis* (ChCl_3_)	189 ± 4.65	22 ± 10.69
